# The influence of natural disasters and multiple natural disasters on self-harm and suicidal behaviour: findings from a nationally representative cohort study of Australian adolescents

**DOI:** 10.1016/j.ssmph.2023.101576

**Published:** 2023-12-09

**Authors:** Ben Edwards, Matthew Taylor, Matthew Gray

**Affiliations:** Centre for Social Research and Methods, Australian National University, Australian Capital Territory, Australia

**Keywords:** suicide, self-harm, suicidal ideation, natural disasters, natural hazards, compound disaster, cascading disaster, recurrent disaster, longitudinal, cohort study

## Abstract

Few studies have examined the relationship between exposure to natural hazards and suicide and self-harm in youth. We extend prior research by investigating the association between multiple disasters and the risks of self-harm and suicide longitudinally in a nationally representative longitudinal cohort of adolescents 14 to 15 years to 18-19 years of age. Natural disasters were identified through parental self-reports for the local area. Different types of multiple disaster exposures were investigated including compound disasters (two or more disasters occurring in the last 12 months), cascading disasters (a disaster that leads to another disaster in the subsequent wave) and consecutive disasters (multiple disasters within the last two years or over an eight-year period). Using 8,714 person-waves of data from 2,908 adolescents, findings from random effect models suggest that parental reports of fire or floods increase the risk of self-harm ideation, self-harm, and suicidal ideation. Compound disasters of fire/flood and drought were also associated with increased risk of suicidal thoughts. Cascading disasters of drought followed by fire/flood increased the risks of self-harm but recurrent consecutive droughts were associated with lower risks of suicidal ideation. Australian adolescents are exposed to high rates of natural disasters that increase the risk of self-harm and thoughts of self-harm and suicide. Climate change will increase risk of natural disaster exposure for all countries. Despite these increased risks, there was resilience to disaster exposure particularly in the case of recurrent drought suggesting that youth, families and communities may well develop protective strategies to support mental health.

## Highlights

• First national cohort study to examine disasters and adolescent suicide.
• Fire or floods increased the risk self-harm, self-harm and suicidal ideation.
• Compound disasters increased the risk of suicidal ideation.
• Cascading disasters increased the risks of self-harm.
• Repeated droughts lowered risks of suicidal ideation.

## 1 Introduction

The long-term impact of natural disasters on self-harm and suicidal behaviour in adolescents are unclear, with varied responses to a range of natural hazards around the globe. For example, studies examining the impacts of earthquakes in Japan have reported an increased risk of children and adolescents’ suicidal attempts, plans and ideation six- and ten-years after the earthquake (Chen, Zhou, Shi, Ma, & Fan, 2020; Tanaka et al., 2016). A Canadian study also reported that suicidal thinking increased in 11 to 19-year olds in the three years following exposure to wildfires (Brown et al., 2021). Another study of the 2004 South-East Asian tsunami on Swedish children aged 10–15 years on holidays compared to a matched comparison group reported that those who were exposed to the tsunami had significantly higher suicidal ideation but not suicide attempts at 18–23 years (Adebäck, Schulman, & Nilsson, 2018). However, to date, the number and type of natural hazards examined has been limited. Most studies have lacked comparison groups and have had small unrepresentative samples of children and youth. Furthermore, there have been even fewer studies of the implications of multiple disasters for suicidal and self-harm behaviours of children and adolescents.

The influence of multiple and co-occurring disasters on self-harm and suicidal thoughts and behaviour of young people is largely unknown. For example, the only study that examines adolescents and multiple disasters used data from telephone calls to a mental health crisis line during the 2020 wildfire season in the United States (Sugg, Runkle, Hajnos, Green, & Michael, 2022). In this study wildfires were not associated with elevated reporting of suicidal thoughts and self-harm (Sugg et al., 2022) but the authors did note that the COVID-19 pandemic could have overshadowed impacts of fires given the already elevated levels of calls to the crisis line. The only study of children examining the impacts of multiple disasters and suicidal ideation reported that the Great East Japan Earthquake and tsunami increased the risk of suicidal ideation for girls who experienced trauma related to an earthquake at preschool age, but not boys (Fujiwara et al., 2017). Exposure to multiple disasters has also been linked to greater lifetime risk of suicide attempts in Australian adults by Reifels, Spittal, Dückers, Mills, and Pirkis (2018). However, their representative cross-sectional survey did not report the link extended to suicidal ideation or plans.

Having a better understanding of the impact of exposure to natural disasters, and particularly multiple disasters, on children and adolescents is becoming increasingly important given the projections that climate change will increase the frequency of natural disasters and that the co-occurrence of natural disasters will become the “new normal” (National Academy of National Academies of Sciences, 2022). Potential mechanisms that may explain how youth mental health could be undermined by exposure to natural disasters include the direct trauma of exposure, a deterioration of parental mental health and reduced parenting capacity, the financial strain on households and on local communities (Hunter, Gray, & Edwards, 2012; Taylor & Edwards, 2022; Conger, Ge, Elder, Lorenz, & Simons, 1994; IPCC, 2022). More broadly, climate change has also been associated with climate anxiety in youth which could have implications for depression, self-harm and suicide (Crandon, Scott, Charlson, & Thomas, 2022; Sanson, Van Hoorn, & Burke, 2019; Scribberas & Fernando, 2022).

This paper examines the impacts of natural disasters on suicidal behaviour and self-harm among Australian youth. It uses parent-reported exposures to natural disasters from the Longitudinal Study of Australian Children (LSAC), a nationally representative cohort study of adolescents followed from ages.14-15 to 18–19. The study has two aims. First, to document the association between exposure to a fire, flood or drought on suicide and self-harm from 14 to 19 years of age. Second, to test whether exposure to compound disasters (two or more disasters occurring in the previous 12 months), cascading disasters (drought followed by fires) and consecutive disasters (multiple disasters within the last two years or over an eight-year period) are associated with suicidal behaviour or self-harm over the course of adolescence. This study extends prior research by including many types of natural hazards, longitudinal data on suicidal behaviour and self-harm over a six-year period, a nationally representative comparison group not exposed to disasters, and different types of multiple disaster exposures.

## 2 Methods

### 2.1 Study design and participants

LSAC, also known as *Growing Up in Australia*, is a longitudinal cohort study of Australian children that began in 2004 (Edwards, 2014). Written informed consent was provided by parents at the Wave 1 interview and this was provided by young people at 14–15 years as well. In this study we use the K-Cohort, born between March 1999 and February 2000, because there were three waves of self-harm and suicidal behaviour information collected via self-administered computerised surveys of adolescents when they were 14–15 to 18–19 years. The parents of these children were first interviewed between March and November 2004 (Mohal et al., 2023). The LSAC sample is a clustered design, based on postcodes, with stratification across capital cities and balance of state for each state and territory from Medicare Australia's enrolment database, considered the most comprehensive database of Australia's population (Edwards, 2014). Medicare is a universal healthcare system that provides government subsidies to all population for primary and secondary care (98% of parents register their children with Medicare by 12 months of age; Soloff, Lawrence, & Johnstone, 2005). LSAC data is publicly available to the scientific community through the Australian Data Archives.

### 2.2 Procedures

The first wave included data on 4983 children in the K-Cohort and the eighth wave of LSAC retains 60.9% of the wave 1 K-Cohort.

Our key outcomes were collected in waves 6 to 8, when the K-Cohort was aged 14–15 to 18–19 years from Computer Assisted Self Interview.

For this study we focus on exposure to natural disasters reported by biological mothers or a parent who knew the child best. The data used is from when the K-Cohort was aged 10–11 to 18–19 years. Thus, disaster exposure includes the period when outcomes were collected, but also collects exposures up to 4 years previous to when suicide and self-harm were first measured. This is an important feature of the design, as it enables cumulative effects of disaster exposure to be estimated. The LSAC Data User Guide provides a detailed description of the study design and procedures (Mohal et al., 2023).

The Australian Institute of Family Studies Ethics Committee provided ethics approval for the LSAC, and all participants provided written informed consent.

### 2.3 Dependent variables

Self-harm: measures of self-harm were derived from the Avon Longitudinal Study of Parents and Children (ALSPAC): Life of a 16+ Teenager questionnaire (ALSPAC, 2007). Young people were asked to respond to Yes or No to the following questions “During the last 12 months have you thought about hurting yourself on purpose in any way (i.e. by taking an overdose of pills, or by cutting or burning yourself)?” and “During the past 12 months have you hurt yourself on purpose in any way (i.e. by taking an overdose of pills, or by cutting or burning yourself)?” This was measured from 14 to 15 years.

Suicide-related behaviour: Measures of suicide-related behaviours were derived from the National Survey of Mental Health & Wellbeing and measured from 14 to 15 years (Australian Bureau of Statistics, 2007). We focussed on suicidal ideation (‘During the past 12 months did you ever consider attempting suicide?“) and attempts (“During the past 12 months, how many times did you actually attempt suicide?“).

### 2.4 Disaster exposures

Exposure to fires, floods and droughts: In this study we used primary caregivers report of exposure to bushfires/floods and droughts in the previous year. Parents were asked whether their home or local area was affected in the last 12 months. Although the suicide and self-harm data are only available from waves 6 to 8, we use disaster exposure data from wave 4 onwards because of our interest in understanding multiple disaster exposures on these outcomes. In this study geospatial data was only available for large geographic areas and therefore caregiver self-reports of disaster were a better measure of disaster exposure given that they were more localised. Moreover, there is some evidence that self-report disaster is more strongly correlated with disaster impacts on household finances (Hunter, Gray, & Edwards, 2012; Edwards, Gray & Borja, 2021a) and child mental health and family functioning was more strongly correlated with caregiver reports of disaster exposure than geospatial measures of disasters (Edwards, Gray & Borja, 2021a). However, geospatial data may well be more appropriate for other studies such as those examining the impacts of smoke from wildfire (Molitor, Mullins, & White, 2023).

### 2.5 Multiple natural disaster exposures

Following Leppold, Gibbs, Block, Reifels, and Quinn (2022), we operationalise compound, cascading and consecutive disasters in this longitudinal cohort (Lawrence et al., 2015; National Academy of Science, 2022).

Compound disasters: Two or more natural disasters that occur simultaneously, operationalised as two or more disasters that occurred in the previous 12-months.

Cascading disasters: Disasters that increase in progression over time and generate unexpected secondary events. In this study we focus on instances where fire follows a drought in the previous wave.

Consecutive disasters: Consecutive disasters are two or more disasters that occur in successive waves of the LSAC survey in the same area. We generate two types of variables. Firstly, the number of disasters in each survey wave for each natural hazard - this includes separate variables for fire/flood and for drought. Secondly, we generate a separate cumulative consecutive measure of disasters for all natural hazards measured - this measure aggregates all disasters up until that survey wave.

### 2.6 Covariates

Statistical models also included child age (in years), child sex at birth, whether the child was born overseas, and whether the child was from an Aboriginal or Torres Strait Islander background. Other covariates included whether the family had moved house since the last wave, whether they lived in a regional or metropolitan area, and their state of residence. Neighbourhood Socio-Economic Advantage/Disadvantage is measured by the Socio-Economic Indexes for Areas which is a standardized score for socioeconomic position by geographic area (postcode of family domicile) compiled from 2011 Australian Census data. We used the Index of Relative Socioeconomic Advantage/Disadvantage to capture neighbourhood advantage and disadvantage, which numerically summarizes the social and economic conditions of Australian neighborhoods (national mean of 1000 and a standard deviation (SD) of 100, where higher values represent greater advantage and less disadvantage).

### 2.7 Statistical analyses

We estimated a random effects model to account for clustering of waves within adolescents. We used the xtlogit command in STATA 15. The following covariates were included: child age, child sex, child Aboriginal or Torres Strait Islander background, whether the family had moved since the last wave, neighbourhood advantage/disadvantage, regional area and state of residence.

For each outcome we estimated five random effects models for self-reported disasters. The first model tests the impact of disasters on outcomes at the same wave. The next four models test the impact of compound, cascading, consecutive and multiple consecutive disasters, respectively. Compound disasters are two disasters that occur in the last 12-months. For cascading disasters, we are testing the impact of a fire/flood occurring at the same wave as the outcome variable that was preceded by a drought in wave t −1. For consecutive disasters we are testing the cumulative influence of a single disaster from wave 4 through to wave 8 (so there is lagged influence as well as a contemporaneous influence of a disaster). Multiple consecutive disasters are the cumulative risk of exposure to multiple disaster types from wave 4 through to 8.

Population attributable fractions are used in public health to understand how important a particular factor is in contributing to ill health. While not assuming a causal effect, the technique provides a ‘thought experiment’ on the degree to which an illness or a natural disaster contributes to rate of ill health in the population (in this case rates of self-harm and suicide ideation and behaviour in Australian youth). To estimate a population attributable fraction for the impact of natural hazards on suicide and self-harm we used the Stata program ‘punafcc’ developed by Roger Newsom using methods recommended by Greenland and Drescher (1993).

### Funding source

This research was supported by the Australian Medical Research Future Fund (APP1201335). The funders had no influence on the research in this paper.

## 3 Results

**Fig. 1:**
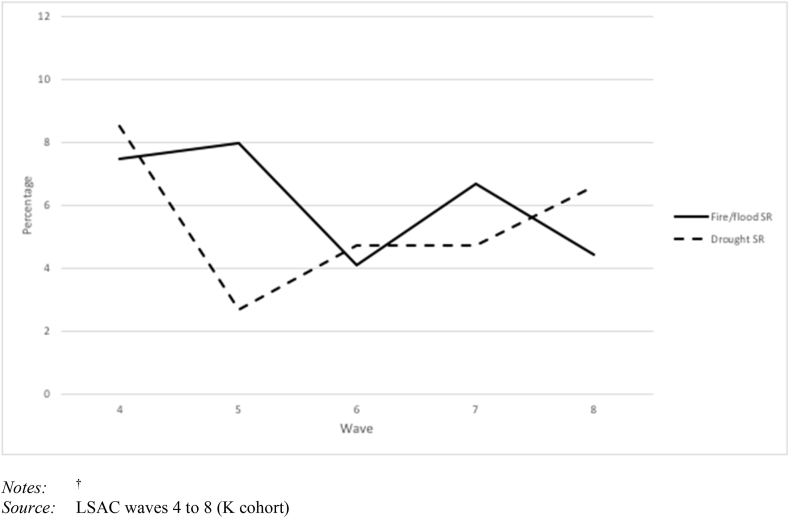
**Percentage of children exposed to disasters by wave**^†^ *Notes:*^†^

*Source:* LSAC waves 4 to 8 (K cohort)

Given that consecutive disaster measures incorporate disaster exposures from the previous waves, we report on disaster exposure in waves 4 to 8. Table 1 shows that on average, 5% of children were directly affected by fire or floods and a further 5% were exposed to drought from wave 4 to 8. However, Fig. 1 shows that there was variation in exposure to fire/floods from wave 4 to 8, with higher levels of exposure in wave 4 and 5 (7–8%) and then in wave 7 (7%). For drought there were fewer peaks over wave 4 to 8 with a peak in wave 4 (9%) and in wave 8 (7%).

**Table 1:** Descriptive statistics for dependent and independent variables, waves 6 to 8.

Variable	Observations	Individuals	%	Mean	SD
*Dependent variables*					
Self-Harm	8709	2903	9·52		
Thoughts of Self-Harm	8708	2903	18·25		
Suicide Attempt	8725	2908	4·60		
Suicide Ideation	8714	2905	10·28		
*Disaster*					
Fire/flood^1^	8725	3582	4·94		
Fire/flood – Consecutive^2^	8709	3580	24.07		
Drought ^1^	8725	3582	5.24		
Drought – Consecutive^2^	8709	3580	20.24		
Compound disaster^1^	8725	3582	1.09		
Cascading disaster – Drought to fire^2^	8507	3498	0.47		
Multiple – Consecutive^2^	8710	3580		5.94	5.46
*Demographic characteristics*					
Age (14–20)	8725	3582		16.29	1.71
Neighbourhood Advantage/Disadvantage	8725	3582		1015·24	75·07
Indigenous	8725	3582	2·01		
Child sex – female (Female = 1; Male = 0)	8725	3582	49·38		
Regional area	8725	3582	38·17		
Moved since last wave	8725	3582	14·01		
State					
New South Wales	8725	3582	30.77		
Victoria	8725	3582	23.01		
Queensland	8725	3582	21.63		
South Australia	8725	3582	6.77		
Western Australia	8725	3582	10.52		
Tasmania	8725	3582	3.50		
Northern Territory	8725	3582	0.91		
Australian Capital Territory	8725	3582	2.89		

Notes: 1. Disasters that occur in the same wave as the outcome variable (waves 6 to 8). This includes singular disasters such as fire, flood or drought and compound disasters where two disasters occur in the same wave.2. Many multiple disasters measure cumulative exposure over more than one wave (consecutive, multiple – consecutive and cascading). This means that they take account all waves of disaster exposure, from the earliest measurement in wave 4.

The suicide and self-harm measures were collected in waves 6 to 8 from 2903 to 2908 children and include 8708 to 8714 person-waves of data. However, cascading disasters and consecutive disaster measures included natural disaster data from wave 4 as well.

Findings from random-effects logistic regression models suggest that fire/floods in the last year were associated with statistically significantly higher rates of self-harm and self-harm ideation and suicidal thoughts (Table 2). Droughts were not associated with self-harm or suicide.

**Table 2:** Natural disasters and self-harm, self-harm ideation, suicidal ideation and suicide attempts.

	Self Harm	95% Confidence Interval	Self-Harm Ideation	95% Confidence Interval	Suicide Attempt	95% Confidence Interval	Suicidal Ideation	95% Confidence Interval
	Odds Ratio		Odds Ratio		Odds Ratio		Odds Ratio	
**Self-report**								
Fire/flood	1.57*	1.04, 2.38	1.64**	1.16, 2.32	1.44	0.84, 2.49	1.61**	1.07, 2.42

Drought	1.22	0.77, 1.92	1.168	0.79, 1.70	1.60	0.90, 2.83	0.99	0.63, 1.58
*N*	8709		8708		8725		8714	

Adjusted for the following variables: child age, child sex, child is a migrant, child is Aboriginal or Torres Strait Islander, moved house since last wave, regional area, state dummy, SEIFA Index of Advantage and Disadvantage. These summarise Table 2a in Supplementary material.+*p* < 0.10, *p < 0.05, **p < 0.01, ***p < 0.001.

**Table 3:** Self-reported multiple natural disasters and self-harm, self-harm ideation, suicidal ideation and suicide attempts.

	Self Harm	95% Confidence Interval	Self-Harm Ideation	95% Confidence Interval	Suicide Attempt	95% Confidence Interval	Suicidal Ideation	95% Confidence Interval
	Odds Ratio		Odds Ratio		Odds Ratio		Odds Ratio	
**Compound**^a^	1.70	0.76, 3.77	1.60	0.79, 3.24	2.20	0.83, 5.88	2.50*	1.16, 5.42
*N*	8709		8708		8725		8714	
**Cascading**^a^								
Drought & Fire/flood	3.36^+^	0.90, 12.43	2.08	0.62, 6.96	0.13	0.01, 1.80	2.02	0.49, 8.25
*N*	8491		8490		8507		8496	
**Consecutive**^b^								
Fire/flood recurrent	1.19	0.93.1.53	1.17	0.94, 1.44	1.09	0.78, 1.51	1.13	0.89, 1.47
Drought recurrent	0.88	0.67, 1.15	0.91	0.73, 1.13	0.87	0.62, 1.23	0.70*	0.53, 0.93
*N*	8710		8709		8726		8715	
**Consecutive**^b^								
Multiple	1.10	0.97, 1.25	1.10	0.99, 1.23	1.10	0.93, 1.29	0.99	0.87, 1.12
*N*	8710		8709		8726		8715	

Adjusted for the following variables: child age, child sex, child is a migrant, child is Aboriginal or Torres Strait Islander, moved house since last wave, regional area, state dummy, SEIFA Index of Advantage and Disadvantage. These summarise Tables 3a–3d in the Appendix.+*p* < 0.10, *p < 0.05, **p < 0.01, ***p < 0.001.

a Disasters that occur in the same wave as the outcome variable (waves 6 to 8). This includes singular disasters such as fire, flood or drought and compound disasters where two disasters occur in the same wave.

b Many multiple disasters measure cumulative exposure over more than one wave (consecutive, multiple – consecutive and cascading). This means that they take account all waves of disaster exposure, from the earliest measurement in wave 4.

To understand how much variation in outcomes could be explained by fire or floods, population attributable fractions were calculated while holding all other covariates equal. Population attributable fractions suggested that eradicating fires or floods would reduce the rates of suicide ideation by 2.0 percentage points (95% CI: 0.54 to 3.84) from a base of 10.3%, self-harm ideation by 1.7 percentage points (95% CI: 0.55 to 2.76) from a base of 18.35 and self-harm by 2.0 percentage points (95% CI: 0.28 to 3.62) from a base of 9.5%.

Multiple exposures to natural disasters based on parent self-reports was significantly associated with suicidal ideation for compound disasters only. In this case, the population attributable fraction suggested that for every compound disaster prevented, there was a reduction of suicidal ideation by 1 percentage point (95%CI: 0.2–1.9%) from a base of 10.3% in the population of Australian youth. Cascading impacts of drought to fire/flood was associated with an increased risk of self-harm (*p* < 0.10). For consecutive disasters that were recurrent over several waves, children exposed to recurrent droughts were at significantly lower risk of suicidal ideation. There was no evidence of consecutive multiple disasters being associated with any self-harm or suicide variables.

## 4 Discussion

In this study we find that sudden onset disasters were associated with increased self-harm ideation, self-harm and suicidal ideation of adolescents. Fire or floods in the last year increased the risk of self-harm, self-harm ideation and suicidal thoughts of adolescents. Exposure to some types of multiple disasters were associated with suicide ideation and self-harm. Compound disasters of fire/flood and drought were also associated with increased risk of adolescents having suicidal thoughts. Cascading disasters of drought followed by fire/flood were associated with increased risks of adolescents’ self-harm but recurrent consecutive droughts were associated with significantly lower risks of suicidal ideation of youth. To understand the potential impacts of natural disasters for the mental health of the population of young people in Australia we also used population attributable fractions. While our estimates are not causal, they provide an upper bound estimate of the capacity to address mental health challenges by eliminating the impacts of disasters entirely. Our findings suggest that eradication of sudden onset disasters could have some substantial benefits for population health. For instance, this would lead to a reduction in suicidal ideation by 2 percentage points from a base rate of 10%.

Findings in the current study are consistent with previous research that have also found disasters were associated with higher levels of suicidal ideation (Brown et al., 2021; Reifels et al., 2018). The only other longitudinal study of youth also showed small increased risks of suicidal ideation in response to fire (Brown et al., 2021). A retrospective cross-sectional study reported much greater lifetime exposure to multiple natural disasters in Australia than in our study (Reifels et al., 2018). In comparison our study reported modest impacts on suicidal ideation and suicide attempts. Differences in findings may suggest that further life time exposures accumulated to create greater risks, or simply reflect a delayed impact on self-harm and suicidal ideation and behaviour of natural disasters. Future research is important to help clarify the relationship between exposure and suicidal ideation and attempts across the lifetime, thereby helping to inform strategies to protect against the mental health consequences associated with natural disasters.

This study provides the first longitudinal analysis of multiple disaster exposure on mental health in adolescents and explicitly testing and operationalising compound, cascading and consecutive disasters for longitudinal studies. The increased risks of cascading disasters for self-harm has not been documented previously, although one other study examining cascading disasters and suicidal behaviour reported greater risks of suicidal ideation for girls in the Great East Japan Earthquake and tsunami (Fujiwara et al., 2017). The varied influence of different types of multiple disasters (compound, cascading, consecutive) on self-harm and suicidal behaviour could be a function of the timing in disaster exposures combined with variable impacts of sudden onset (fire/flood) and slow onset disasters. For example, in this study sudden onset multiple disasters led to increased risks when disasters were relatively recent (compound disasters or cascading disasters). However, there were no increased risks for recurrent sudden onset disasters and lower risks of suicidal ideation with recurrent drought (a slow onset disaster). Perhaps exposure to a slow onset chronic stressor such as drought that can span several years (the Millennium drought in Australia was from 2001 to 2009) may lead to habituation to the stressor or the mustering of community and family resilience to improve wellbeing in the long term (Masten, 2021). An important element of adaption and resilience to future multiple disasters will be to identify factors that protect against poor mental health and build resilience. Prior research suggests that strengthening financial supports to disaster will offset the negative consequences for parental mental health and parenting (Conger et al., 1994), but that family, schools and communities all may play a role in supporting young people's mental health (Masten, 2021). For public policy and government responses, local community engagement has been considered to be an important element in any successful response to multiple disasters (Leppold et al., 2022). Future research focused on identifying protective factors are warranted, as well as further replication in other contexts.

Despite its strengths, there were some limitations of the current study. The random effects regressions likely reflect associations rather than robust causal estimates. However, testing multiple exposures to disasters over time meant that fixed effects models could not be estimated (McNeish & Kelley, 2019). Given that the disaster exposure could be considered to be a random occurrence, there is some support for the causality of our estimates. There were also some limitations in the measurement of outcomes and disaster exposures. Like many studies, the items measuring suicidal thoughts and behaviour do not attempt to strictly define a suicide attempt and there is a risk that self-harm is conflated with suicidal thoughts and behaviours (Ammerman, Burke, Jacobucci, & McClure, 2021). This risk is limited in LSAC as self-harm items were asked first, and then suicide questions second. Comparative national studies, like the Australian Child and Adolescent Survey of Mental Health and Wellbeing (Lawrence et al., 2015) generated similar rates, with rates of suicidal ideation, at 8%, a little lower than in LSAC, and the rates self-harm consistent between the two studies. Another potential limitation in our measures is the risk of under-reporting disaster exposure. Caregivers were asked to report on disaster exposure in the last year but waves of data collection occur every two years thereby increasing the risk of under-reporting. Despite this limitation, the current study is an advance on retrospective reporting of multiple natural disasters which suffer from retrospective recall bias (Galea, Maxwell, & Norris, 2008).

## 5 Conclusions

The 2019–20 wildfires attracted world attention (author et al., 2020) and highlighted that Australia has a high risk of disaster exposure. However, the 2023 wildfires in North America underscore that our findings are increasingly applicable to other countries. Beyond wildfires, the IPCC predicts that there are increased risks of drought in Australia but also in South America, the Mediterranean, China, North America and Eurasia (IPCC, 2022). Despite our study findings, it is important to note there was considerable resilience to disaster exposure suggesting that youth, families and communities may well develop protective strategies to support mental health. Further research to identify these protective factors is urgently needed to inform better disaster mitigation policy and service responses (NASEM, 2022).

## Funding source

This research was supported by the Australian Medical Research Future Fund (MRF1201355). The funders had no influence on the research in this paper.

## Competing interests

The authors declare that they have no competing interest.

## Ethical statement

The Australian Institute of Family Studies Ethics Committee provided ethics approval for the LSAC, and all participants provided written informed consent.

## Declaration of competing interest

The authors declare that they have no known competing financial interests or personal relationships that could have appeared to influence the work reported in this paper.

The authors declare the following financial interests/personal relationships which may be considered as potential competing interests.

## Data Availability

LSAC data is available from the Australian Data Archives
