# Supplementary material for: The influence of natural disasters and multiple natural disasters on self-harm and suicidal behaviour: findings from a nationally representative cohort study of Australian adolescents
Source: SSM Popul Health. 2023 Dec 9;25:101576. doi: 10.1016/j.ssmph.2023.101576 (PMC10788300; doi:10.1016/j.ssmph.2023.101576)
Supplement: Multimedia component 1 [file mmc1.docx]

**Table 2a**

Logistic regression models of the effects of disasters (self-report) on self-harm and suicide

|  | **Self-harm** | | **Harm ideation** | | **Suicide** | | **Suicidal ideation** | |
| --- | --- | --- | --- | --- | --- | --- | --- | --- |
|  | **OR** | **95% CI** | **OR** | **95% CI** | **OR** | **95% CI** | **OR** | **95% CI** |
| Fire/flood | 1.57 | 1.04, 2.38 | 1.64 | 1.16, 2.32 | 1.44 | 0.84, 2.49 | 1.61 | 1.07, 2.42 |
| Drought | 1.22 | 0.77, 1.92 | 1.16 | 0.79, 1.7 | 1.60 | 0.9, 2.83 | 0.99 | 0.63, 1.58 |
| Moved house | 1.41 | 1.08, 1.83 | 1.38 | 1.11, 1.72 | 2.22 | 1.59, 3.12 | 1.41 | 1.08, 1.82 |
| Age | 0.98 | 0.93, 1.03 | 1.05 | 1, 1.09 | 1.02 | 0.95, 1.09 | 1.10 | 1.05, 1.16 |
| Immigrant | 1.13 | 0.63, 2.05 | 1.07 | 0.64, 1.79 | 0.73 | 0.31, 1.74 | 1.04 | 0.57, 1.91 |
| Indigenous | 0.94 | 0.41, 2.16 | 0.95 | 0.47, 1.93 | 1.51 | 0.57, 4.01 | 0.66 | 0.28, 1.60 |
| Child sex – female (Female = 1; Male = 0) | 3.77 | 2.91, 4.87 | 3.95 | 3.18, 4.91 | 1.76 | 1.28, 2.42 | 1.94 | 1.52, 2.48 |
| Region | 0.70 | 0.53, 0.92 | 0.80 | 0.63, 1.01 | 0.55 | 0.38, 0.79 | 0.72 | 0.55, 0.95 |
| Neighborhood Advantage/ Disadvantage | 1.00 | 1.00, 1.00 | 1.00 | 1.00, 1.00 | 1.00 | 0.99, 1.00 | 1.00 | 1.00, 1.00 |
| Victoria | 0.92 | 0.66, 1.27 | 0.97 | 0.73, 1.29 | 0.85 | 0.55, 1.32 | 0.87 | 0.63, 1.22 |
| Queensland | 0.88 | 0.63, 1.23 | 0.91 | 0.68, 1.21 | 0.85 | 0.55, 1.32 | 0.93 | 0.67, 1.30 |
| South Australia | 1.62 | 1.01, 2.60 | 1.40 | 0.92, 2.14 | 1.37 | 0.74, 2.53 | 1.42 | 0.88, 2.30 |
| Western Australia | 0.99 | 0.64, 1.51 | 1.18 | 0.82, 1.70 | 0.81 | 0.45, 1.46 | 0.85 | 0.55, 1.31 |
| Tasmania | 0.72 | 0.37, 1.44 | 0.94 | 0.53, 1.67 | 0.97 | 0.42, 2.27 | 0.98 | 0.51, 1.90 |
| Northern Territory | 0.87 | 0.24, 3.10 | 2.51 | 0.98, 6.43 | 0.57 | 0.09, 3.79 | 1.59 | 0.51, 5.01 |
| Australian Capital Territory | 1.55 | 0.79, 3.03 | 1.52 | 0.85, 2.72 | 0.84 | 0.31, 2.33 | 1.50 | 0.76, 2.97 |
| Intercept | 3.77 | 3.00, 4.72 | 3.94 | 3.30, 4.70 | 4.63 | 3.37, 6.36 | 4.25 | 3.44, 5.25 |
| *N* | 8709 |  | 8708 |  | 8725 |  | 8714 |  |

**Table 3a**

Logistic regression models of the effects of compound disasters (self-report) on self-harm and suicide

|  | **Self-harm** | | **Harm ideation** | | **Suicide** | | **Suicidal ideation** | |
| --- | --- | --- | --- | --- | --- | --- | --- | --- |
|  | **OR** | **95% CI** | **OR** | **95% CI** | **OR** | **95% CI** | **OR** | **95% CI** |
| Compound | 1.70 | 0.76, 3.77 | 1.60 | 0.79, 3.24 | 2.20 | 0.83, 5.88 | 2.50 | 1.16, 5.42 |
| Moved house | 1.41 | 1.08, 1.83 | 1.38 | 1.11, 1.72 | 2.23 | 1.59, 3.12 | 1.38 | 1.06, 1.79 |
| Age | 0.98 | 0.93, 1.03 | 1.05 | 1, 1.09 | 1.02 | 0.95, 1.09 | 1.10 | 1.05, 1.16 |
| Immigrant | 1.13 | 0.62, 2.03 | 1.06 | 0.63, 1.77 | 0.73 | 0.31, 1.73 | 1.04 | 0.56, 1.90 |
| Indigenous | 0.93 | 0.41, 2.15 | 0.95 | 0.47, 1.92 | 1.52 | 0.57, 4.01 | 0.65 | 0.27, 1.57 |
| Child sex – female (Female = 1; Male = 0) | 3.75 | 2.91, 4.85 | 3.94 | 3.17, 4.89 | 1.75 | 1.27, 2.41 | 1.94 | 1.51, 2.47 |
| Region | 0.72 | 0.55, 0.95 | 0.83 | 0.66, 1.05 | 0.59 | 0.41, 0.84 | 0.73 | 0.56, 0.96 |
| Neighborhood Advantage/ Disadvantage | 1.00 | 1.00, 1.00 | 1.00 | 1.00, 1.00 | 1.00 | 0.99, 1.00 | 1.00 | 1.00, 1.00 |
| Victoria | 0.90 | 0.65, 1.25 | 0.95 | 0.72, 1.26 | 0.83 | 0.54, 1.29 | 0.86 | 0.62, 1.20 |
| Queensland | 0.86 | 0.62, 1.21 | 0.89 | 0.67, 1.19 | 0.83 | 0.54, 1.29 | 0.92 | 0.66, 1.29 |
| South Australia | 1.59 | 0.99, 2.55 | 1.38 | 0.91, 2.11 | 1.34 | 0.73, 2.48 | 1.41 | 0.87, 2.28 |
| Western Australia | 0.97 | 0.63, 1.48 | 1.16 | 0.81, 1.67 | 0.79 | 0.44, 1.42 | 0.84 | 0.54, 1.30 |
| Tasmania | 0.72 | 0.36, 1.43 | 0.95 | 0.53, 1.68 | 0.94 | 0.41, 2.20 | 0.99 | 0.51, 1.92 |
| Northern Territory | 0.85 | 0.24, 3.05 | 2.47 | 0.97, 6.31 | 0.56 | 0.08, 3.69 | 1.58 | 0.50, 4.94 |
| Australian Capital Territory | 1.53 | 0.78, 3.01 | 1.51 | 0.85, 2.71 | 0.83 | 0.30, 2.31 | 1.49 | 0.75, 2.95 |
| Intercept | 3.75 | 2.99, 4.71 | 3.93 | 3.29, 4.69 | 4.61 | 3.36, 6.33 | 4.26 | 3.45, 5.26 |
| *N* | 8709 |  | 8708 |  | 8725 |  | 8714 |  |

**Table 3b**

Logistic regression models of the effects of consecutive disasters (self-report) on self-harm and suicide

|  | **Self-harm** | | **Harm ideation** | | **Suicide** | | **Suicidal ideation** | |
| --- | --- | --- | --- | --- | --- | --- | --- | --- |
|  | **OR** | **95% CI** | **OR** | **95% CI** | **OR** | **95% CI** | **OR** | **95% CI** |
| Fire/flood - Consecutive | 1.19 | 0.93, 1.53 | 1.17 | 0.94, 1.44 | 1.09 | 0.78, 1.51 | 1.14 | 0.89, 1.47 |
| Drought - Consecutive | 0.88 | 0.67, 1.15 | 0.91 | 0.73, 1.13 | 0.87 | 0.62, 1.23 | 0.70 | 0.53, 0.93 |
| Fire/flood | 1.33 | 0.83, 2.15 | 1.43 | 0.96, 2.12 | 1.33 | 0.71, 2.48 | 1.41 | 0.89, 2.26 |
| Drought | 1.42 | 0.79, 2.55 | 1.30 | 0.80, 2.10 | 1.92 | 0.91, 4.05 | 1.58 | 0.87, 2.87 |
| Moved house | 1.40 | 1.08, 1.83 | 1.38 | 1.10, 1.72 | 2.22 | 1.58, 3.12 | 1.41 | 1.09, 1.83 |
| Age | 0.98 | 0.93, 1.03 | 1.04 | 1.00, 1.09 | 1.02 | 0.95, 1.1 | 1.11 | 1.05, 1.17 |
| Immigrant | 1.15 | 0.64, 2.08 | 1.08 | 0.65, 1.81 | 0.73 | 0.31, 1.75 | 1.04 | 0.56, 1.91 |
| Indigenous | 0.94 | 0.41, 2.16 | 0.95 | 0.47, 1.93 | 1.54 | 0.58, 4.08 | 0.69 | 0.29, 1.65 |
| Child sex – female (Female = 1; Male = 0) | 3.77 | 2.92, 4.87 | 3.96 | 3.18, 4.92 | 1.76 | 1.28, 2.43 | 1.95 | 1.52, 2.49 |
| Region | 0.69 | 0.52, 0.92 | 0.79 | 0.62, 1.01 | 0.56 | 0.38, 0.81 | 0.75 | 0.56, 1.00 |
| Neighborhood Advantage/ Disadvantage | 1.00 | 1.00, 1.00 | 1.00 | 1.00, 1.00 | 1.00 | 0.99, 1.00 | 1.00 | 1.00, 1.00 |
| Victoria | 0.92 | 0.66, 1.27 | 0.97 | 0.73, 1.29 | 0.86 | 0.55, 1.32 | 0.88 | 0.63, 1.22 |
| Queensland | 0.86 | 0.62, 1.21 | 0.9 | 0.67, 1.20 | 0.84 | 0.54, 1.3 | 0.91 | 0.65, 1.27 |
| South Australia | 1.63 | 1.02, 2.62 | 1.41 | 0.93, 2.16 | 1.38 | 0.74, 2.54 | 1.43 | 0.88, 2.32 |
| Western Australia | 0.97 | 0.63, 1.48 | 1.16 | 0.81, 1.67 | 0.80 | 0.44, 1.44 | 0.82 | 0.53, 1.27 |
| Tasmania | 0.73 | 0.37, 1.44 | 0.94 | 0.53, 1.68 | 0.96 | 0.41, 2.25 | 0.95 | 0.49, 1.85 |
| Northern Territory | 0.88 | 0.25, 3.15 | 2.54 | 0.99, 6.50 | 0.57 | 0.09, 3.81 | 1.58 | 0.50, 4.99 |
| Australian Capital Territory | 1.57 | 0.8, 3.08 | 1.54 | 0.86, 2.75 | 0.86 | 0.31, 2.38 | 1.54 | 0.78, 3.04 |
| Intercept | 3.76 | 3.00, 4.72 | 3.94 | 3.30, 4.70 | 4.64 | 3.38, 6.37 | 4.26 | 3.45, 5.25 |
| *N* | 8709 |  | 8708 |  | 8725 |  | 8714 |  |

**Table 3c**

Logistic regression models of the effects of cascading disasters (self-report) on self-harm and suicide

|  | **Self-harm** | | **Harm ideation** | | **Suicide** | | **Suicidal ideation** | |
| --- | --- | --- | --- | --- | --- | --- | --- | --- |
|  | **OR** | **95% CI** | **OR** | **95% CI** | **OR** | **95% CI** | **OR** | **95% CI** |
| Cascading | 3.36 | 0.91, 12.43 | 2.08 | 0.62, 6.96 | 0.13 | 0.01, 1.80 | 2.02 | 0.49, 8.25 |
| Fire or flood self-reported | 1.32 | 0.84, 2.07 | 1.49 | 1.03, 2.15 | 1.46 | 0.82, 2.61 | 1.43 | 0.93, 2.22 |
| Drought self-reported | 1.08 | 0.68, 1.74 | 1.09 | 0.74, 1.61 | 1.51 | 0.84, 2.73 | 0.94 | 0.58, 1.51 |
| Fire or flood reported previous wave | 1.09 | 0.73, 1.64 | 1.05 | 0.75, 1.47 | 1.14 | 0.67, 1.96 | 1.05 | 0.70, 1.58 |
| Drought reported previous wave | 1.07 | 0.61, 1.87 | 0.93 | 0.58, 1.49 | 1.19 | 0.59, 2.41 | 0.85 | 0.47, 1.52 |
| Moved House | 1.38 | 1.05, 1.81 | 1.34 | 1.07, 1.68 | 2.14 | 1.51, 3.03 | 1.39 | 1.06, 1.81 |
| Age | 0.98 | 0.93, 1.04 | 1.05 | 1.01, 1.10 | 1.01 | 0.94, 1.09 | 1.11 | 1.05, 1.17 |
| Immigrant | 1.17 | 0.64, 2.13 | 1.03 | 0.61, 1.75 | 0.77 | 0.32, 1.87 | 1.00 | 0.54, 1.88 |
| Indigenous | 0.92 | 0.39, 2.2 | 0.96 | 0.46, 2.02 | 1.65 | 0.61, 4.51 | 0.66 | 0.26, 1.65 |
| Child sex – female (Female = 1; Male = 0) | 3.72 | 2.87, 4.84 | 4.05 | 3.24, 5.06 | 1.79 | 1.29, 2.48 | 1.92 | 1.50, 2.47 |
| Region | 0.71 | 0.53, 0.94 | 0.81 | 0.64, 1.04 | 0.57 | 0.39, 0.83 | 0.73 | 0.55, 0.98 |
| Neighborhood Advantage/ Disadvantage | 1.00 | 1.00, 1.00 | 1.00 | 1.00, 1.00 | 1.00 | 0.99, 1.00 | 1.00 | 1.00, 1.00 |
| Victoria | 0.90 | 0.65, 1.27 | 0.98 | 0.73, 1.3 | 0.85 | 0.54, 1.33 | 0.86 | 0.61, 1.22 |
| Queensland | 0.88 | 0.63, 1.24 | 0.92 | 0.69, 1.24 | 0.85 | 0.54, 1.34 | 0.93 | 0.66, 1.31 |
| South Australia | 1.58 | 0.98, 2.55 | 1.41 | 0.92, 2.17 | 1.41 | 0.76, 2.64 | 1.44 | 0.88, 2.35 |
| Western Australia | 0.99 | 0.65, 1.53 | 1.19 | 0.82, 1.73 | 0.85 | 0.47, 1.55 | 0.89 | 0.57, 1.39 |
| Tasmania | 0.76 | 0.38, 1.51 | 0.99 | 0.55, 1.77 | 1.00 | 0.42, 2.37 | 1.03 | 0.52, 2.01 |
| Northern Territory | 0.96 | 0.26, 3.53 | 2.62 | 0.98, 7.00 | 0.63 | 0.09, 4.32 | 1.45 | 0.43, 4.88 |
| Australian Capital Territory | 1.56 | 0.79, 3.08 | 1.60 | 0.89, 2.89 | 0.90 | 0.32, 2.53 | 1.59 | 0.80, 3.18 |
| Intercept | 3.84 | 3.05, 4.83 | 4.06 | 3.39, 4.85 | 4.79 | 3.47, 6.60 | 4.38 | 3.54, 5.42 |
| *N* | 8491 |  | 8490 |  | 8507 |  | 8496 |  |

**Table 3d**

Logistic regression models of the effects of consecutive multiple disasters (self-report) on self-harm and suicide

|  | **Self-harm** | | **Harm ideation** | | **Suicide** | | **Suicidal ideation** | |
| --- | --- | --- | --- | --- | --- | --- | --- | --- |
|  | **OR** | **95% CI** | **OR** | **95% CI** | **OR** | **95% CI** | **OR** | **95% CI** |
| Consecutive Multiple | 1.10 | 0.97, 1.25 | 1.10 | 0.99, 1.23 | 1.10 | 0.93, 1.29 | 0.99 | 0.87, 1.12 |
| Moved house | 1.41 | 1.08, 1.83 | 1.37 | 1.10, 1.71 | 2.26 | 1.61, 3.16 | 1.41 | 1.09, 1.83 |
| Age | 0.98 | 0.93, 1.03 | 1.04 | 1.00, 1.09 | 1.02 | 0.94, 1.09 | 1.11 | 1.05, 1.17 |
| Immigrant | 1.15 | 0.63, 2.07 | 1.08 | 0.65, 1.80 | 0.73 | 0.31, 1.75 | 1.03 | 0.56, 1.89 |
| Indigenous | 0.93 | 0.40, 2.14 | 0.94 | 0.46, 1.89 | 1.50 | 0.57, 3.99 | 0.66 | 0.28, 1.60 |
| Child sex – female (Female = 1; Male = 0) | 3.75 | 2.90, 4.85 | 3.93 | 3.16, 4.88 | 1.75 | 1.27, 2.41 | 1.94 | 1.51, 2.47 |
| Region | 0.69 | 0.52, 0.92 | 0.79 | 0.62, 1.01 | 0.56 | 0.39, 0.81 | 0.75 | 0.57, 1.00 |
| Neighborhood Advantage/Disadvantage | 1.00 | 1.00, 1.00 | 1.00 | 1.00, 1.00 | 1.00 | 0.99, 1.00 | 1.00 | 1.00, 1.00 |
| Victoria | 0.90 | 0.65, 1.25 | 0.95 | 0.72, 1.26 | 0.83 | 0.54, 1.29 | 0.86 | 0.62, 1.20 |
| Queensland | 0.87 | 0.62, 1.22 | 0.9 | 0.68, 1.2 | 0.84 | 0.54, 1.3 | 0.92 | 0.66, 1.28 |
| South Australia | 1.61 | 1.01, 2.58 | 1.4 | 0.92, 2.13 | 1.35 | 0.73, 2.5 | 1.40 | 0.86, 2.27 |
| Western Australia | 0.97 | 0.64, 1.49 | 1.17 | 0.82, 1.68 | 0.79 | 0.44, 1.42 | 0.83 | 0.54, 1.29 |
| Tasmania | 0.75 | 0.38, 1.48 | 0.98 | 0.55, 1.74 | 0.97 | 0.42, 2.28 | 0.98 | 0.51, 1.91 |
| Northern Territory | 0.87 | 0.24, 3.12 | 2.52 | 0.99, 6.44 | 0.57 | 0.09, 3.79 | 1.57 | 0.5, 4.93 |
| Australian Capital Territory | 1.55 | 0.79, 3.04 | 1.52 | 0.85, 2.72 | 0.83 | 0.3, 2.32 | 1.51 | 0.77, 2.99 |
| Intercept | 3.77 | 3.01, 4.73 | 3.93 | 3.29, 4.69 | 4.64 | 3.38, 6.38 | 4.27 | 3.46, 5.27 |
| *N* | 8710 |  | 8709 |  | 8726 |  | 8715 |  |
